# Enhancing children's health literacy: a scoping review of resources for ‘Curriculum for Wales’ health and well-being design, implementation, and assessment

**DOI:** 10.1093/heapro/daaf225

**Published:** 2025-12-19

**Authors:** Levi Hughes, Michaela James, Helen Lewis, Gisselle Tur Porres, Helen Yu, Emily Marchant

**Affiliations:** National Centre for Population Health and Wellbeing Research, Population Data Science, Medical School, Faculty of Medicine, Health and Life Science, Swansea University, Swansea SA2 8PP, United Kingdom; National Centre for Population Health and Wellbeing Research, Population Data Science, Medical School, Faculty of Medicine, Health and Life Science, Swansea University, Swansea SA2 8PP, United Kingdom; Centre for Research into Practice, Department of Education and Childhood Studies, Faculty of Humanities and Social Sciences, Swansea University, Swansea SA2 8PP, United Kingdom; Centre for Research into Practice, Department of Education and Childhood Studies, Faculty of Humanities and Social Sciences, Swansea University, Swansea SA2 8PP, United Kingdom; Value-Based Health and Care Academy, School of Management, Faculty of Humanities and Social Sciences, Swansea University, Swansea SA1 8PP, United Kingdom; Centre for Research into Practice, Department of Education and Childhood Studies, Faculty of Humanities and Social Sciences, Swansea University, Swansea SA2 8PP, United Kingdom

**Keywords:** health literacy, curriculum, schools, resources, curriculum design, curriculum for wales, health and well-being, area of learning and experience, education

## Abstract

Health literacy (HL) plays an important role in developing the skills and capacities to make health-enhancing decisions, impacting health and well-being. Primary schools are key settings for developing HL through the reformed ‘Curriculum for Wales’ (CfW) and its ‘Health and Well-being Area of Learning and Experience’ (H&WB AoLE). With school-level autonomy offered in CfW design, resources are fundamental for curriculum design, implementation, and assessment. As the CfW is in its infancy, the visibility and quality of resources available to schools is unclear. This scoping review aimed to identify resources publicly available to primary schools to enable the design, implementation and assessment of the H&WB AoLE. A search was conducted across academic databases and sources of grey literature. Twelve sources (grey literature: *n* = 7, peer-reviewed research: *n* = 5) were selected for inclusion and discussed as a descriptive overview. The identified resources highlight a gap between policy intentions of the CfW framework and how this is implemented in practice. However, there is potential to address these concerns through self-assessment tools, collaborative improvement, planning, and evidence-informed practice. The broad nature of the CfW framework and variation in the availability and quality of health-related resources informing CfW design may result in variability in learning opportunities, influencing how children’s HL is developed. Prioritizing HL as a core CfW learning outcome could streamline the translation of broad CfW guidance into impactful design, implementation and assessment of the H&WB AoLE.

Contribution to health promotion statement

- First scoping review of its kind to explore the resources available to primary schools in designing, implementing, and assessing the ‘Health and Well-being Area of Learning and Experience’ within the recent implementation of the reformed ‘Curriculum for Wales’.
- Proposes three recommendations for impacting the translation of policy into practice for teaching, learning, and assessment. This includes the integration of health literacy as a learning process and outcome, prioritizing developmental opportunities for current and future educational practitioners to maximize their role as curriculum designers, and strengthening co-production between schools and researchers.

## Introduction

Good health and well-being during childhood is essential to develop the foundations that maximize achievement and prospects throughout the life course (Clark *et al*. 2020). Health literacy (HL) is a key and modifiable determinant of health that empowers children to develop the skills to make positive and informed health-promoting decisions, and is fundamental to achieving good health and well-being (Baccolini *et al*. 2021). Multiple definitions exist worldwide, including the World Health Organization’s (WHO) definition as the ability of individuals to ‘access, understand, appraise and use information and services in ways that promote and maintain good health and well-being’ (WHO 2024). In the context of Wales, UK, HL is defined as ‘the ability and motivation of an individual to access, understand, communicate and evaluate narrative and numeric information to promote, manage and improve their health status throughout their lifetime’ (Puntoni 2010, p.1). Middle childhood (age 7–11) is a key period where children begin to establish competencies, behaviours, and HL-related skills that track into adolescence and adulthood (Otten *et al*. 2022). During this period, it is important to provide opportunities to develop children’s HL through the domains proposed by Nutbeam (2000). This includes progressing from basic cognitive skills understanding health-related information (functional HL), to more advanced skills in interpreting and applying health-related information (interactive HL) and finally through critically analysing and using health-related information (critical HL).

HL was identified as a priority in Wales in 2010, though progress in the field has stalled since (Puntoni 2010). However, recent wider education and public health developments, coupled with a policy and strategy shift from the Welsh Government which reflect the importance of HL have been observed (Marchant and Crick 2023, 2024, Marchant 2024). Most notably, this includes ‘Successful Futures: Independent Review of Curriculum and Assessment Arrangements’, presenting to Welsh Government the urgent need for curriculum reform to meet the needs of an evolving society (Donaldson 2015). In response, the reformed ‘Curriculum for Wales’ (CfW), with renewed statutory focus on health and well-being, was implemented into school settings for children aged 3–16 on a phased basis from September 2022 (Welsh Government 2022a).

The CfW is underpinned by ‘Four Purposes’ that provide a starting point for every child and young person in Wales, one of which is ‘healthy, confident individuals’ (Welsh Government 2022a). The framework for teaching and learning is structured through six ‘Areas of Learning and Experience’ (AoLE) (Welsh Government 2020). This includes the ‘Health and Well-being AoLE’ (H&WB) that encompasses the importance of good health and well-being through a holistic approach to active citizenship (Welsh Government 2020). Each AoLE is guided by ‘Statements of What Matters’ and for the ‘H&WB AoLE’; this includes ‘developing physical health and well-being has lifelong benefits’ (Welsh Government n.d.-c). Learning progression through the H&WB AoLE requires development of HL skills through the conceptual domains of ‘functional’, ‘interactive’, and ‘critical’ HL (Nutbeam 2000). Thus, whilst HL is not explicitly mentioned within the CfW framework, it is an important learning process and outcome of the CfW, and aligns with the five-component framework of HL as a learning process and outcome, proposed by Paakkari and Paakkari (2012). The framework consists of the learning conditions required to develop HL in schools; ‘theoretical knowledge’, ‘practical knowledge’, ‘critical thinking’, ‘self-awareness’, and ‘citizenship’, and has significant cross-over with the framework, guidance and overarching aims within the CfW.

As research suggests that schools are key spaces in facilitating the development of HL skills (Kickbusch 2008, Paakkari *et al*. 2019, Otten *et al*. 2022), targeting primary schools (age 3–11) as key settings has the potential to enhance HL. Renewed statutory focus on health in the CfW provides opportunities to strengthen the health and well-being of current and future generations (Okan *et al*. 2023, Marchant and Crick 2024). Within the CfW, school-level autonomy is offered to educational practitioners in the design, implementation and assessment of the CfW framework and its application in practice. However, while evidence supports this approach with greater professional autonomy embedded (Donaldson 2015), early research exploring its implementation has highlighted several challenges (Power *et al*. 2020). For example, increased professional autonomy may lead to variation in practice and thus, different educational experiences and outcomes for children across Wales (Power *et al*. 2020). This is at odds with the Welsh Government’s national mission of high standards and reducing the inequality gap (Welsh Government 2017). There is a risk that a flexible curriculum framework could amplify these disparities (Marchant and Crick 2024).

To better understand how best to support primary schools, it is essential to explore the space between policy intent, curriculum guidance and enacting this in practice to develop all children as ‘healthy, confident individuals’. This is particularly important when considering the H&WB AoLE, as limited professional learning and a lack of opportunities for continued professional development (CPD) specific to health and well-being may contribute to gaps in professional knowledge and confidence (Marchant and Crick 2024). In the light of these challenges, access to resources has been identified as fundamental to support schools in the design, implementation and assessment of the H&WB AoLE and wider CfW framework (Welsh Government 2024). Resources include policy documents and guidance, digital platforms or websites, evaluation or self-assessment tools, and evidence-based or peer-reviewed research. Given the infancy of the CfW, it is unclear what resources are currently available, including formal or informal sources such as grey literature and academic research (Walker *et al*. 2019, Welsh Government 2021). Therefore, this scoping review, guided by the five-stage framework proposed by Arksey and O’Malley (2005) aims to identify and provide a descriptive overview of the resources available online for educational practitioners in the design, implementation and assessment of the CfW H&WB AoLE.

## Materials and methods

### Review design

This scoping review was conducted by a research team from the ‘National Centre for Population Health’ (CPH) and the ‘Centre for Research into Practice’ (CenPrac) at Swansea University to explore existing guidance, literature, and research relating to the H&WB AoLE, wider CfW, and HL. Given the infancy of the CfW implementation in practice, limited research is emerging in this field and therefore, given this newer area of study a scoping review was most suitable. This study was guided by the work of Arksey and O’Malley (2005) who propose scoping review approaches with the purpose to ‘summarize and disseminate research findings’ (p. 3) by describing the findings in more detail from a range of research within the field in order to inform ‘policy makers, practitioners and consumers who might otherwise lack time or resources to undertake such work themselves’ (p. 3). In this context, this was applied to the recently implemented CfW, with specific focus on the H&WB AoLE. Therefore, the methodology followed the five stages recommended by Arksey and O’Malley (2005); research question, identification, study selection, charting data, and collating and summarizing results. The review was guided by the Preferred Reporting Items for Systematic Reviews and Meta-Analyses extension for scoping reviews (PRISMA ScR; Tricco *et al*. 2018), presented in Supplementary File A. The scoping review protocol was not registered. This scoping review did not involve human participants and therefore did not require ethical approval.

#### Stage 1: identifying the research question

The research team, whose expertise spans children’s health, well-being, education, HL, curriculum development, and implementation, conducted brainstorming activities. This included literature searching and analysis, critical discussion, and drawing on professional experience to identify current gaps in research and generate the research question:

- What resources are publicly available online for educational practitioners to support the design, implementation, and assessment of the CfW H&WB AoLE?

#### Stage 2: identifying relevant studies

To identify relevant and quality resources, a comprehensive independent search by L.H. (November 2024) and M.J. (December 2024) was conducted across multiple platforms. Firstly, a search for academic research was conducted across five electronic databases (Education Resources Information Centre, Sage Journals, Science Direct, Taylor and Francis Online, and Wiley Online Library) and three publishers (British Education Research Association Educational Research Journals, BMC Public Health, and The Lancet). Secondly, a search was conducted in Google to identify grey literature.

The search terms used across all platforms included: ‘children’ OR ‘primary school aged children’ OR ‘learners’ AND ‘health’ OR ‘health and well-being’ OR ‘health and wellbeing’ OR ‘well-being’ OR ‘wellbeing’ OR ‘health literacy’ OR ‘physical health’ OR ‘physical development’ OR ‘mental health’ or ‘social and emotional wellbeing’ AND ‘Curriculum for Wales’ OR ‘new curriculum in Wales’ OR ‘curriculum reform in Wales’ OR ‘health and wellbeing area of learning and experience’ OR ‘health and wellbeing AoLE’ OR ‘Four Purposes Wales’ OR ‘health education Wales’ OR ‘successful futures’ AND ‘new curriculum resources Wales’ OR ‘new curriculum toolkits Wales’ OR ‘curriculum guidance Wales’ OR ‘Curriculum for Wales framework’ OR ‘health and wellbeing AoLE resources’ OR ‘health and wellbeing AoLE guidance’ OR ‘health and wellbeing AoLE framework’. All sources were last searched or consulted on 16 December 2024.

#### Stage 3: study selection

The inclusion criteria for the scoping review were designed to ensure relevant and quality resources aligned to the core aim of this study published within the last 5 years (2019–24). Grey literature sources were included in this scoping review if they were developed to support primary schools in the design, implementation and assessment of the H&WB AoLE or to facilitate the development of HL skills. This included professional resources produced by the Welsh Government and organizations with focus on health and well-being. Academic research exploring the H&WB AoLE and the CfW more broadly was included if it involved qualitative studies, mixed methods studies, or literature reviews published between 2020 and 2024. The eligible studies focused on the following participants: primary school teaching staff in Wales, including head teachers, deputy head teachers, classroom teachers, and Additional Learning Needs Coordinators as well as stakeholders such as policy leads from the Welsh Government and representatives from Higher Education Institutions (HEIs). For this scoping review, all staff are referred to hereon as educational practitioners.

Grey literature sources were excluded if they did not directly relate to the CfW (e.g. the previous ‘National Curriculum in Wales’ (2008–22)) or if they were outside the dates of this study (2019–24). Academic research was excluded if it consisted of dissertations, studies based on secondary schools or studies that provided findings from international contexts.

#### Stage 4: charting the data

**Figure 1:**
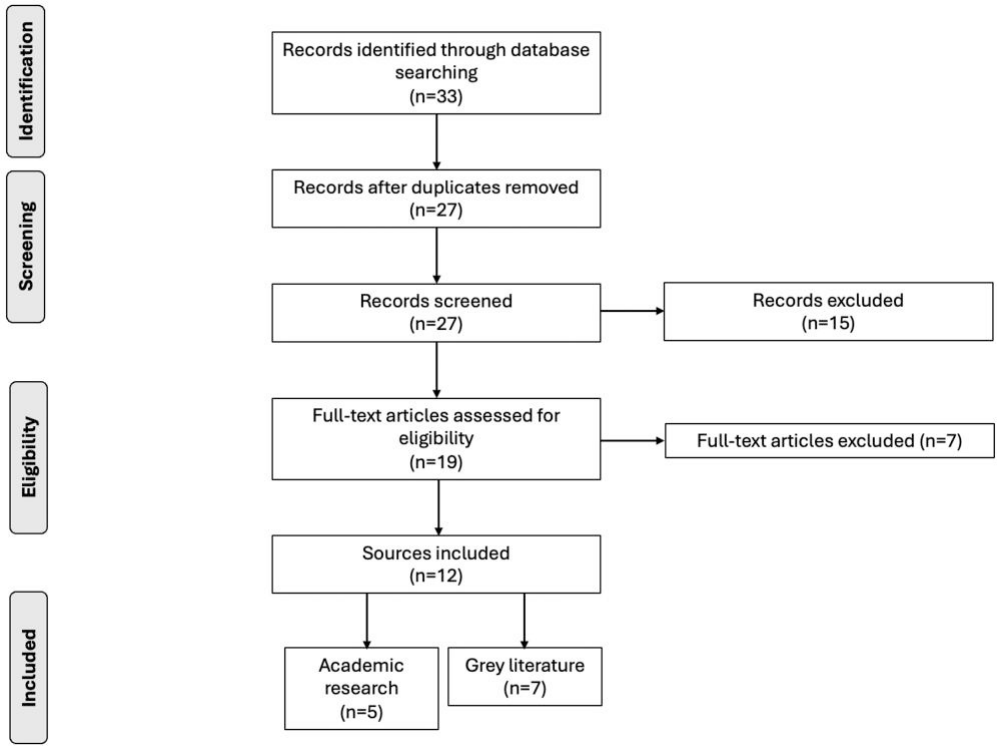
PRISMA flow diagram of scoping review process.

The initial search produced a total of 33 sources that met the search terms. After removing 6 duplicate entries, 27 sources remained for full text screening and were imported to Rayyan (Ouzzani *et al*. 2016). This assessed aspects such as study design, sample size, data collection methods, data analysis, and potential biases. Following this process, a further 15 studies were excluded for not meeting the inclusion criteria and 12 sources were used in total (*n* = 7 grey literature, *n* = 5 academic research) and stored on Mendeley (n.d.). This process also involved EM as a third researcher who provided validation of selected sources, and any conflicts were resolved through a group discussion. There was very high agreement between researchers. A PRISMA flow diagram depicting the scoping review process is presented in Figure 1.

#### Stage 5: collating, summarizing, and reporting the results

The collation, synthesis, and analysis of selected sources involved organizing key themes within the grey literature and academic research that aligned with the objectives of this scoping review. The collation and presentation of sources aimed to address the objective of identifying resources that are publicly available online for educational practitioners. A critical appraisal was conducted to ensure that all included sources were relevant, credible and aligned with the inclusion and exclusion criteria. This appraisal was applied across both academic and grey literature with clearly defined inclusion and exclusion criteria, a comprehensive search strategy using appropriate databases and grey literature platforms, relevant search terms and consideration for potential risks of bias during title, abstract, and full text screening. Subsequently, the extracted data were organized into categories to provide a clear and descriptive summary of the findings. Rather than seeking comparability across sources, this scoping review aimed to discuss thematic patterns and key concepts as a descriptive overview across included studies specific to the design, implementation and assessment of the CfW H&WB AoLE. Thus, the robustness and relevance of sources were considered during the identification, selection and interpretation of sources.

## Results

### Overview

This scoping review aimed to identify and provide a descriptive overview of the resources publicly available online that support primary schools to design, implement, and assess the H&WB AoLE and in turn, develop children’s HL. In total, 12 sources were identified, with a breakdown of these sources presented in Table 1.

**Table 1:** Overview of identified sources.

Author(s)/source	Year of publication	Type (Academic/Grey)	Overview of key themes/findings
Welsh Government	2020	Grey literature	Government curriculum framework that provides statutory guidance and overarching curriculum framework for teaching and learning within the ‘Curriculum for Wales Health and Well-being Area of Learning and Experience’. This includes its aims to develop learners as healthy, confident individuals who can manage their physical, mental, and emotional well-being throughout life. The guidance promotes learning-centred approaches, ensuring progression and assessment are embedded throughout, presented by curriculum components including ‘Statements of What Matters’, ‘Descriptions of Learning’, and ‘Principles of Progression’.
O’Prey *et al*.	2019	Grey literature	This report summarizes an independent analysis of responses from over 1600 educational stakeholders (practitioners, school leaders, parents, special interest groups and public sector organizations), providing feedback on the draft Curriculum for Wales 2022. Feedback on the ‘Health and Well-being Area of Learning and Experience’ was generally positive, but concerns were raised regarding clarity in guidance and implementation, progression and assessment.
Welsh Government	2024	Grey literature	A non-statutory guide for anyone involved in the development and production of resources and materials in support of design of learning and teaching within the ‘Curriculum for Wales’. The resources aim to support practitioners’ pedagogical approaches for curriculum design.
Hwb	n.d.	Grey literature	Curriculum design guidance hosted through the National Digital Learning Platform for Wales, sharing online resources to enhance teaching and learning and support design of the ‘Curriculum for Wales’. Resources are categorized across each of the six ‘Areas of Learning and Experience’, including ‘Health and Well-being’. These can be filtered according to a number of tailored sub-groups, including learning progression stage (Progression Steps 1–5), age, audience, assessment, and skills.
Adnodd	n.d.	Grey literature.	Curriculum design resource bank hosted through Welsh Government commissioned organization, Adnodd. Adnodd works with a range of suppliers to commission adaptable and accessible resources that support ‘Curriculum for Wales’ design. These include bilingual interactive tools, revision guides and teacher packs. Whilst resource development is achieved through a commissioning process, all digital resources are publicly available to teachers through Adnodd and Hwb.
Central South Consortia (CSC, regional education consortia)	n.d.	Grey literature	One of four regional education bodies, working cross-regionally across 9 themes with focus on the ‘Curriculum for Wales’. This includes professional learning to enhance pedagogy and practice, and supporting the development and realization of the curriculum. CSC provides high-quality, evidence-informed professional learning, support and resources related to curriculum design, progression, teaching and assessment. This includes those specific to ‘Health and Well-being’ across three themes: Food and Nutrition, Physical Activity, and Social, Emotional and Mental Well-being.
Margerison and Ravenscroft	2020	Academic research	Discusses how online character education tools can be integrated into school curricula to enhance citizenship education and personal development, using the Draft Curriculum for Wales 2022 as an example. A five-component framework is proposed (Margerison C-Model), which emphasizes five aspects of character development through practical, technology-enabled applications. With a focus on digital tools, this offers strategies for translating broad educational policies into actionable classroom practices that promote identity formation and social-emotional growth.
Stirrup *et al*.	2024	Academic research	The authors examine how the ‘Curriculum for Wales’ re-legitimizes health and physical education (HPE) messages within its ‘Health and Well-being Area of Learning and Experience’. This includes the shift toward competency-based, holistic approaches that prioritize well-being, over traditional performance-driven models. The paper highlights the greater teacher autonomy to implement HPE content and the ‘Health and Wellbeing’ guidance, encouraging flexibility and learner-centred practices. However, the authors acknowledge challenges relating to performativity and individual responsibility, advocating for ongoing professional development to ensure these progressive curriculum aims and translation of broad guidance into practice.
Conn and Hutt	2020	Academic research	The paper emphasizes how the **‘**Health and Well-Being Area of Learning and Experience’ within the ‘Curriculum for Wales’ plays a critical role in supporting learners with ALN. It highlights that the curriculum’s emphasis on inclusive practice, holistic development, learner autonomy, and ‘stage not age’ progression can be beneficial for learners with ALN. Through interviews with four educational practitioners within the Welsh education system, findings highlight concerns about practical challenges, including meeting the needs of learners with complex difficulties and ensuring teacher capacity for inclusive practice. The authors argue the tensions of aspirational policy and broad guidance, proposing that professional development and clear articulation of teachers as agents of change are critical for realizing a truly inclusive ‘Health and Well-being’ curriculum.
Hughes and Lewis	2020	Academic research	The paper investigates how the ‘Health and Well-Being Area of Learning and Experience’ was enacted in a Pioneer primary school. Through a case study using a mixed methods approach, the research collected a combination of survey data from over 600 teachers across 80 schools and semi-structured interviews with seven staff members. Findings from teachers highlighted positive feedback regarding ‘Health and Well-being's’ emphasis on mental health and emotional resilience, but that due to uncertainty and workload they relied on ‘off-the-peg’ resources. This highlights the tensions between the reform’s aim of teacher autonomy and the practical realities of curriculum implementation. Findings show that pre-packaged schemes provided confidence and structure, but sustainability and depth of professional learning remained challenges. The authors conclude that autonomy requires ongoing support, clear guidance, and time for teachers to develop expertise and adapt approaches flexibly to learners’ need.

The CfW H&WB AoLE guidance (Welsh Government 2020) is one of the most fundamental resources for all schools in Wales, providing the overarching curriculum framework for teaching and learning. The five ‘Statements of What Matters’ for the H&WB AoLE focus on opportunities for children to develop their understanding and competence around health:

- Developing physical health and well-being has lifelong benefits.
- How we process and respond to our experiences affects our mental health and emotional well-being.
- Our decision-making impacts on the quality of our lives and the lives others.
- How we engage with social influences shapes who we are and affects our health and well-being.
- Healthy relationships are fundamental to our well-being.

The ‘Principles of Progression’ define what progress means across the H&WB AoLE and the ‘Descriptions of Learning’ outline how this progression occurs across each ‘Statement of What Matters’, represented through advancement through five ‘Progression steps’. These represent the importance of enhancing HL skills and children gaining autonomy over their own health and well-being. Central to achieving these is how HL is developed through Nutbeam’s domains of ‘functional’, ‘interactive’. and ‘critical HL’ (Nutbeam 2000). For example, the ‘Principles of Progression’ include deepening understanding and application of health-related information, including: ‘develop conceptual knowledge and critical understanding in a range of aspects of health and well-being and personal behaviour’ towards ‘progress from primarily considering themselves, to considering others, both in their own relationships with others and in wider local, national and international contexts’. A central component of the CfW is school-level curriculum design, enabling educational practitioners to create learning experiences for the H&WB AoLE (encompassing the ‘Statements of What Matters’, ‘Principles of Progression’. and ‘Descriptions of Learning’) tailored to the health, well-being and HL needs of children.

The ‘Curriculum for Wales 2022 Feedback and Analysis’ (O’prey *et al*. 2019) presents the findings from an independent evaluation of responses on the draft CfW 2022. This was conducted through a consultation process consisting of a qualitative survey (online via email, by post and in person) and regional events, workshops and focus groups. A total of 1680 responses were received between May and July 2019 from a wide range of education stakeholders, including primary school and secondary school educational practitioners, school leaders, parents, special interest groups, public sector organizations and children and young people.

The responses highlighted important considerations relevant to the quality of health and well-being curriculum design and developing HL in primary schools across Wales. Key themes identified were the broad guidance and the increased autonomy placed on schools, further suggesting the potential that exists in varied interpretations of the ‘Statements of What Matters’, ‘Principles of Progression’, and ‘Descriptions of Learning’. For example, there was a concern that such variability could lead to divergence in the content, coherence and quality of health-related pedagogy, ultimately impacting the development of children’s HL. It was recommended by educational practitioners based in primary schools that more explicit signposting of relevant H&WB AoLE resources would help to address these issues.

The ‘Resources and Supporting Materials Guide’ (Welsh Government 2024) was produced by the Welsh Government, consisting of seven key principles that outline what schools and organizations should consider and avoid when developing or adapting resources. These principles can facilitate primary schools to engage with relevant and reliable resources that support and enhance their design, implementation and assessment of the H&WB AoLE and in turn, provide quality and equitable opportunities for the development of HL. What to consider included consistency across the CfW, clear learning rationales, flexibility in teaching and learning, embedding research-informed pedagogical approaches, the importance of co-production, supporting an understanding of the Welsh language and inclusivity. Factors to avoid included resources that are based on isolated activities that do not relate to or build upon prior learning, and resources that are designed to be used on a one-off or ‘off-the-shelf’ basis. In this context, ‘off-the-shelf’ resources for the H&WB AoLE refer to materials designed by organizations with an interest in health and well-being. They are advertised to primary schools as time-saving tools that comprise of predetermined learning objectives, classroom activities, assessment methods and specific time allocations and therefore, aim to reduce efforts on curriculum building and lesson planning. The Welsh Government provides examples of what good resources look like on Hwb, the National Digital Learning Platform for Wales (Welsh Government n.d.-a).

Hwb hosts a variety of online resources that aim to enhance teaching and learning and support CfW design (Welsh Government n.d.-a). The online resources provide equal and free access to content that is commissioned by the Welsh Government, designed by educational practitioners and organizations and underpinned by the Welsh Government’s ‘Resources and Supporting Materials Guide’. For the design, implementation and assessment of the H&WB AoLE, examples of these resources include the Public Health Wales toolkits and the Welsh Ambulance’s ‘Blue Light Hub’ App.

The Welsh Government has also recently introduced a new professional learning platform within Hwb that enables educational practitioners to search for materials that meet their professional learning needs (Welsh Government n.d.-b). Examples of the resources include enquiry projects conducted by primary schools across Wales that aim to improve health-related pedagogy and equity, provision maps on how to approach teaching and learning about healthy relationships and training modules on the significance of misinformation online. In addition, the professional learning area on Hwb provides primary school practitioners with access to initiatives such as the ‘Well-being Partnership Programme’ which consists of well-being leads from schools across Wales working collaboratively to respond to the well-being needs of schools and settings.

Both sets of resources on Hwb, for teaching/learning and professional learning, are relevant to the H&WB AoLE and the enhancement of children’s HL. They support the role of educational practitioners within the CfW as curriculum designers as opposed to providing them with universal products to adopt. In greater detail, the resources for teaching and learning can be used by educational practitioners to engage children in discussions specific to HL skills regarding social influences and managing risks, safety and coping in challenging situations. In addition, the resources for professional learning demonstrate examples of best practice and aim to develop leadership and management skills in health and well-being, thereby developing educational practitioners’ health-related knowledge and thus, enhancing confidence and competence in pedagogy.

‘Adnodd’ is an online educational resource organization that aims to provide schools with CfW design and implementation resources (Adnodd n.d.). Commissioned by the Welsh Government, Adnodd will facilitate the co-production of resources across key partnerships. This includes collaboration between the Welsh Government, educational practitioners and organizations to maximize the potential of funding, professional knowledge and innovation. The resources established by Adnodd will be hosted on Hwb. This decision was made to streamline educational practitioners’ access to relevant and reliable materials that are based on the priorities and needs of schools, children, their families and local communities (O’prey *et al*. 2019).

‘Central South Consortium’ (CSC) is one of four school improvement services in Wales that works in partnership with school leaders, local authorities and the Welsh Government to improve educational outcomes. The other services include ‘Educational Achievement Service’, in South East Wales, GwE in North Wales and ‘Partneriaeth’ in Swansea and Carmarthenshire (Central South Consortium Joint Education Service n.d., Educational Achievement Service n.d., GwE n.d., Partneriaeth n.d.). The school improvement services extend the work of the Welsh Government by providing a range of online resources that support primary schools to design, implement and assess the H&WB AoLE that can develop children’s HL (Central South Consortium Joint Education Service n.d.). These resources include pre-recorded or live webinars and support sessions focused on teaching health-promoting behaviours and health and well-being planning at a local level. This is also supplemented through direct collaboration with schools across their regions. For the H&WB AoLE and the development of children’s HL, examples of work carried out could be enquiry projects, gather school-based data and fund school-to-school sessions that enable educational practitioners to share best practice.

Margerison and Ravenscroft (2020) explored how character education can be applied by educational practitioners to achieve the requirements of the CfW, focusing specifically on how learning from lived experiences demonstrates clear links to the H&WB AoLE and ‘Four Purposes’. For example, its opportunities for learning are rooted in real-world contexts that enable children to make connections and apply knowledge. Organizations such as ‘Amazing People Schools’ have resources that support educational practitioners to enact character education in practice (Amazing People Schools n.d.). This includes differentiated materials that encourage children to write stories, engage in role-play or conduct interviews with their peers about their chosen character. These activities can also support children in constructing and shaping their own beliefs and values in relation to the H&WB AoLE.

Stirrup and colleagues (Stirrup *et al*. 2024) proposed an approach that, focuses on embedding pedagogical strategies into everyday teaching practices (Stirrup *et al*. 2024). Their work explored how competency and performance pedagogic models can be implemented by educational practitioners to enhance health education within curriculum reform in Wales. Competency pedagogic models provide children with greater control over the selection, sequencing, and pace of the curriculum (Stirrup *et al*. 2024). Such an approach ensures that learning experiences are relevant and meaningful to children, thereby encouraging their participation in group discussions, structured activities and in turn, movement from ‘functional’, ‘interactive’, to ‘critical’ HL (Nutbeam 2000). Alongside these strengths, the authors (Stirrup *et al*. 2024) also argued how competency pedagogic models, in their full effect, may raise concerns regarding health inequalities as not all children will have the level of health-related knowledge or experience required to make decisions on what and how they learn. For instance, some children within the H&WB AoLE will require the level of scaffolding and support that is available through performance pedagogical models and therefore, it is important that educational practitioners adopt a balanced approach.

Conn and Hutt (2020) explored the impact of the CfW on children with additional learning needs (ALN). Their qualitative study involved interviews with eight participants selected for their expertise on the CfW and ‘ALN Act’. The sample included four participants from Pioneer schools, a group of 68 schools across Wales who were selected to work in collaboration with the Welsh Government and key stakeholders to co-develop and trial the CfW. The sample included an ALN Coordinator and a Professional Learning Lead from a primary school and four participants in stakeholder roles including ALN policy leads at the Welsh Government and regional ALN coordinators. The insights gathered from the participants highlighted how the less prescriptive nature of the CfW has been beneficial for children with ALN who often learn through multiple pathways (Conn and Hutt 2020). In particular, the findings encourage how the broad ‘Statements of What Matters’, the ‘Progression Steps’, and ‘Descriptions of Learning’ enable children with ALN to work at stage, as opposed to age, within the H&WB AoLE, thereby supporting these individuals to develop HL in a way that makes sense to them. The participants also offered key recommendations for maximizing the achievement outcomes of children with ALN, focusing on how educational practitioners should utilize resources that are differentiated not different, skill-based and scaffolded (Conn and Hutt 2020). This can develop and enhance HL through a process of demonstration, modelling and reflection.

Hughes and Lewis (Hughes and Lewis 2020) used ‘off-the-shelf’ resources, materials that are designed organizations with an interest in health and well-being as an example of how primary schools have responded to the challenges of curriculum reform in Wales. There are conflicting perspectives on the nature of ‘off-the-shelf’ resources (Welsh Government 2024). Whilst the Welsh Government suggests that ‘off-the-shelf’ resources limit the shift of educational practitioners from curriculum consumers to curriculum designers (Welsh Government 2024), educational practitioners emphasize how ‘off-the-shelf’ resources provide solutions to workload challenges and offer the foundations for curriculum planning (Hughes and Lewis 2020). For example, rather than limiting professional autonomy and creativity, they can support the design of lesson content and structure, and improve professional knowledge and confidence relating to the H&WB AoLE.

These tensions were identified and explored through qualitative research with a primary school in South Wales. Semi-structured interviews were conducted with seven educational practitioners, of which the sample included two senior leaders, four classroom teachers and one teaching assistant (Hughes and Lewis 2020). This sample of participants is particularly important as their primary school was part of the Pioneer network, schools across Wales who worked in collaboration with the Welsh Government and key stakeholders to co-develop and trial the CfW. Thus, Pioneer schools were among the most informed regarding the design, implementation and assessment of the H&WB AoLE (Hughes and Lewis 2020). Within this context, the participants, despite acknowledging the Welsh Government’s recommendation to avoid ‘off-the-shelf’ resources, perceived that additional support was required in order to successfully embed the H&WB AoLE into practice (Margerison and Ravenscroft 2020). Lastly, the study conducted by Hughes and Lewis formed part of a larger study where Power *et al*. (2020) administered a survey to all Pioneer schools across Wales. There were 634 responses across 81 Pioneer schools and the survey findings revealed how a further 65% of Pioneer schools had adopted an ‘off-the-shelf’ resource to support their design, implementation, and assessment of the CfW (Power *et al*. 2020).

## Discussion

The CfW provides renewed statutory focus on health and well-being through the overarching ‘Four Purposes’, including developing ‘healthy’, ‘confident individuals’, and ‘Health and Well-being’ as one of six AoLEs in which teaching and learning is structured (Welsh Government 2020, 2022a). This school-level curriculum with attention to H&WB offers an opportunity to support children’s HL, a modifiable determinant of health that influences health outcomes throughout the life course (Okan *et al*. 2023). This scoping review aimed to identify resources relevant to the design, implementation, and assessment of the H&WB AoLE process. As the implementation of the CfW is in its infancy (rolled out on a staggered approach since September 2022), this scoping review has highlighted the limited publicly available resources, frameworks, and approaches specific to health and well-being curriculum design for educational practitioners. Through a descriptive overview, these resources demonstrate broad but limited guidance for H&WB design, thus there is a risk of a gap between policy intent (i.e. curriculum framework) and practice (how this is interpreted and implemented in schools). This is important as whilst developing children’s HL is a powerful tool in reducing inequalities and improving health and well-being outcomes, there is a risk that a flexible curriculum framework could amplify these disparities (Marchant and Crick 2024). Based on these resources, this scoping review proposes common themes for further discussion and recommendation for policy design and practice. These are (i) streamlining broad CfW guidance by framing HL as a learning process and outcome of the H&WB AoLE, (ii) how increased professional autonomy for educational practitioners to develop their own pedagogical approaches places barriers on tailored resource development, and (iii) how evidence-informed practice could address these challenges.

### Challenges to educational practitioners’ autonomy and resource development

The ‘Successful Futures: Independent Review of Curriculum and Assessment Arrangements in Wales’ demonstrated a strong desire amongst the educational community for all educational practitioners to have greater autonomy within the national curriculum framework (Donaldson 2015). This recommendation was taken forward by key stakeholders who acknowledged the positive relationship between increased professional autonomy and the likelihood of improved practitioner-retention rates, job satisfaction and student performance outcomes (Jerrim *et al*. 2023). However, whilst some educational practitioners welcomed this approach, others believed that the opportunity to make more school-level decisions within a common framework has instead translated into a lack of detail and depth (O’prey *et al*. 2019, Power *et al*. 2020). Thus, one such proposal is to align the broad H&WB framework to the five-component framework for HL as a learning process and outcome.

Furthermore, for school and classroom-level autonomy, the importance of health-specific CPD opportunities has been considered. CPD is essential for ongoing professional learning in relation to how schools design, implement and assess the H&WB AoLE and wider CfW, supporting the shift of educational practitioners as consumers to curriculum designers (Jones 2011, Davies *et al*. 2018, O’prey *et al*. 2019, Welsh Government 2024). However, the expectation for educational practitioners to respond to curriculum reform with provision that reflects their increased autonomy has been coupled with a lack of investment of CPD opportunities that support enacting policy into impactful practice (O’prey *et al*. 2019).

For health-related professional learning in Wales, this has the potential for unintended consequences, including widening inequalities which is at odds with the Welsh Government’s National Mission (Welsh Government 2017). In addition to the limited opportunities for CPD, the confidence of educational practitioners to act as curriculum designers rests largely on the efficiency of initial teacher education (ITE) in Wales (Estyn 2022, Welsh Government 2022b). ITE is the starting point for training that instils and equips student teachers as future educational practitioners with the necessary knowledge, skills and attitudes. This provides a foundation for student teachers to effectively design, implement and assess the H&WB AoLE (Estyn 2022, Welsh Government 2022b), impacting HL. Currently, there is a dearth of research that explores educational practitioners’ preparedness, including the time allocated to health and well-being and HL during ITE in Wales (Marchant and Crick 2024). Research is urgently required with student and current educational practitioners at varying stages of their career to explore their knowledge and confidence in developing HL and the H&WB AoLE (Marchant and Crick 2024).

This ambiguity creates potential for variation in the quality of teaching and learning, influencing how HL is developed and subsequent health and well-being outcomes throughout the life course. To address these concerns, this scoping review proposes the consideration of HL to be recognized as a core competency within the CfW. This could be achieved through the work of Paakkari and Paakkari (2012) by conceptualizing HL as a learning process and outcome for schools within curriculum guidance. For example, their interpretation of HL provides a framework that could be embedded into the H&WB AoLE to streamline its requirements, emphasizing five core components of theoretical, practical and critical knowledge, self-awareness and citizenship.

### Evidence-informed practice

Given the current limited opportunities for CPD in Wales and the research gap concerning the time allocated to the H&WB AoLE and HL during ITE, supporting primary schools through evidence-informed practice is paramount. Evidence-informed practice refers to the application of high-quality research integrated with professional expertise and it is recognized for its ability to navigate challenges in education and to bridge the gaps in professional learning (Nelson and Campbell 2017, Ramot and Bialik 2023). This scoping review has identified several sources of evidence-informed practice that can guide primary schools in selecting appropriate resources for the design, implementation and assessment of the H&WB AoLE (Conn and Hutt 2020, Hughes and Lewis 2020, Margerison and Ravenscroft 2020, Power *et al*. 2020, Stirrup *et al*. 2024). However, the reach, distribution and impact of this work within schools is limited as academic research has only a small to moderate impact on the interests and decision-making processes of educational practitioners (Vanderlinde and van Braak 2010). Reasons for this include time and funding constraints that restrict schools’ capacity to interpret and embed research findings and data into practice (Vanderlinde and van Braak 2010). Educational practitioners’ engagement with evidence-informed practice has improved over time and primary schools in Wales are now more likely to use and undertake action research in comparison to their counterparts in other UK nations (Smith and Horton 2017, Welsh Government 2021).

Furthermore, Hughes and Lewis (2020) highlighted best-practice examples of using ‘off-the-shelf’ resources, those designed by organizations with an interest in health and well-being but not promoted on Welsh Government platforms. Practitioners in their study commented on the benefits of these resources in addressing workload challenges and uncertainty in enacting curriculum guidance into practice. However, this is at odds with guidance by Welsh Government who suggest avoid using resources that are designed to be used on a one-off or ‘off-the-shelf’ basis. Based on practitioner perspectives, these resources could in fact offer a foundation for co-developed, adaptable materials that balance autonomy with professional collaborations. We suggest that future research should examine how such resources could be co-produced with educators and organizations to ensure alignment with the CfW.

Emphasis should be placed on co-production efforts as collaboration between schools and HEIs would facilitate the production and knowledge translation of further evidence. This can inform practice and develop resources relevant to the H&WB AoLE and HL aligned to educational practitioners’ experiences of identifying problems, evaluating practice, constructing solutions and raising standards.

### Health literacy as a learning process and outcome of the Curriculum for Wales

Although HL is not explicitly mentioned in the current H&WB AoLE or CfW guidance, its principles are represented throughout. For instance, progression through the continuum of learning within the H&WB AoLE reflects the model of HL proposed by Nutbeam (2000), from ‘functional’, ‘interactive’, to ‘critical’ HL. Therefore, adopting the framework for HL as a learning outcome could address the lack of detailed guidance and ensure greater consistency in the content, coherence and quality of the H&WB AoLE across Wales. Such structure can not only streamline broad guidance but also provide a consistent structure that maintains educational practitioners’ professional autonomy. Indeed, the integration of HL within existing curricula has been observed worldwide, including in Finland where it is framed through the five-component framework of Paakkari and Paakkari (2012). Data from the WHO suggests that children in Finland are among some of the most informed about health in Europe (World Health Organization 2019). Thus, in the early phases of curriculum reform, implementation and refinement, we should consider global case studies such as these best-practice case studies in Finland.

### Strengths and limitations

This is the first scoping review to explore the resources available to primary schools in designing, implementing and assessing the H&WB AoLE within the CfW. As the CfW implementation remains in its infancy, this research highlights several resource-specific gaps and opportunities for enhancing the implementation of the CfW, enacting effective and impactful school-level H&WB curriculum design and in achieving one of the ‘Four Purposes’ of developing ‘healthy, confident individuals’. The findings in this scoping review should be considered within limitations present. This includes the resources that the research team had access to. Many resources, especially those designed specifically for educational practitioners, are only accessible to educational practitioners. Thus, it is likely that existing resources for the design, implementation and assessment of the H&WB AoLE and the development of children’s HL were not identified during the search strategy and included within the scoping review. Future research would benefit from exploring resources available specifically to educational practitioners, for example those accessible through Welsh Government services including Hwb and Adnodd. In addition, qualitative research with educational practitioners could gather professional perspectives regarding a wider range of resources used, and how these guide curriculum design, implementation and assessment.

## Conclusion

Given the infancy of the CfW, the scoping review provides valuable insights to inform current and emerging policy and to refine resource provision and practice. This has included three key recommendations:

- The integration of HL as a learning process and outcome into the H&WB AoLE guidance to streamline the design, implementation and assessment of the H&WB AoLE. This can enhance how HL is integrated into teaching and learning and enable equitable approaches to developing the HL of current and future generations.
- Increase opportunities in ITE and CPD that demonstrate how educational practitioners can apply their increased autonomy and flexibility to maximize their role as curriculum designers with specific attention to the H&WB AoLE.
- Strengthen co-production between primary schools and researchers. As there is a willingness amongst schools to increase their engagement with evidence-informed practice, co-production efforts are essential for resources, both professionally-developed and ‘off-the-shelf’, that are based on relevant and reliable data collection methods, lived experiences and classroom realities.

The next phase of this study aims to bridge the gap between research, policy and practice working in collaboration with a sample of primary schools across south Wales to explore their views on the H&WB AoLE and opportunities for enhancing HL.

## Supplementary Material

daaf225_Supplementary_Data

## Data Availability

Data within this scoping review was obtained only from publicly available data in the form of previously published material.
